# Ectodysplasin A1 Deficiency Leads to Osteopetrosis-like Changes in Bones of the Skull Associated with Diminished Osteoclastic Activity

**DOI:** 10.3390/ijms232012189

**Published:** 2022-10-13

**Authors:** Christine Schweikl, Sigrun Maier-Wohlfart, Holm Schneider, Jung Park

**Affiliations:** 1 Department of Pediatrics, University Hospital Erlangen, Friedrich-Alexander University Erlangen-Nürnberg, 91054 Erlangen, Germany; 2 Center for Ectodermal Dysplasias, University Hospital Erlangen, Friedrich-Alexander University Erlangen-Nürnberg, 91054 Erlangen, Germany

**Keywords:** bone, ectodysplasin A1, ectodermal dysplasia, osteopetrosis, osteoclast differentiation, NF-κB, NFAT

## Abstract

Pathogenic variants of the gene *Eda* cause X-linked hypohidrotic ectodermal dysplasia (XLHED), which is characterized by structural abnormalities or lack of ectodermal appendages. Signs of dysplasia are not restricted to derivatives of the ectodermal layer, but mesodermal abnormalities, such as craniofacial dysmorphism, are also frequently observed, suggesting close reciprocal interactions between the ectoderm and mesoderm; however, a causal link has remained unsubstantiated. We investigated the functional impact of defective ectodysplasin A1 (Eda1) signaling on postnatal bone homeostasis in Eda1-deficient Tabby mice. Interestingly, Eda1 was detected in wild-type mouse calvariae throughout postnatal lifetime. In calvariae, bone-lining Osterix (Osx)+ osteoblasts stained positive for Eda1, and osteoclasts were revealed as Eda receptor (Edar)-positive. Moreover, adult Eda1-deficient calvarial bone showed osteopetrosis-like changes with significantly diminished marrow space, which was maintained during adulthood. Concomitantly with osteopetrosis-like changes, Tabby calvarial bone and Tabby bone marrow-derived osteoclasts had far less osteoclastic activity-associated co-enzymes including cathepsin K, Mmp9, Trap, and Tcirg1 (V-type proton ATPase a3 subunit) compared with wild-type calvariae in vivo or osteoclasts in vitro, indicating that Eda1 deficiency may affect the activity of osteoclasts. Finally, we confirmed that nuclear Nfatc1-positive osteoclasts were strongly diminished during mature osteoclastic differentiation under M-CSF and RANKL in the Tabby model, while Fc-EDA treatment of Tabby-derived osteoclasts significantly increased nuclear translocation of Nfatc1. Furthermore, we identified enhanced Nfatc1 and NF-κB transcriptional activity following Fc-EDA treatment in vitro using luciferase assays. Overall, the results indicate that diminished expressions of osteoclastic activity-associated co-enzymes may lead to disturbed bone homeostasis in Tabby calvariae postnatally.

## 1. Introduction

For the human transmembrane protein ectodysplasin A, encoded by the X-chromosomal ectodysplasin A gene (*EDA*; MIM *300451; NM_001399), two different functional isoforms are known to interact with distinct receptors: EDA1 with EDAR and EDA2 with EDA2R (XEDAR) [1,2,3,4]. Pathogenic variants of the gene *EDA* cause X-linked hypohidrotic ectodermal dysplasia (XLHED), a hereditary disease characterized by defective development of ectodermal appendages and a symptomatic triad consisting of hypotrichosis, oligo- or anodontia, and hypo- or anhidrosis [5,6,7,8]. Particularly in infancy, XLHED is a life-threatening condition, as absent or severely reduced sweating may lead to hyperthermia. Due to the deficient development of nasal, tracheal, and bronchial glands, patients also have a higher risk of respiratory infections [5,9].

Various strategies to treat XLHED with an EDA1 replacement protein have been investigated [10,11,12]. Postnatal treatment was partially effective in animal models but not in human patients [13], whereas prenatal administration of a recombinant EDA1 molecule, Fc-EDA, to affected boys resulted in normal sweat gland endowment and sweating ability, development of more teeth, and normalized function of salivary glands [13,14] and, thus, showed the potential to permanently resolve the most relevant clinical problems associated with XLHED [15].

EDA1 is a member of the tumor necrosis factor (TNF) family and is active mainly during development. In the early stages of embryonic development, *EDA*/*EDAR* are known to exhibit a focally restricted expression pattern in developing epithelia. So far, it is uncertain which biological function EDA1 might have postnatally in organs not derived from the embryonic ectoderm. 

Although XLHED is characterized by absence or abnormalities of ectodermal appendages [16,17], abnormal tissues of mesodermal origin are also found in XLHED patients, indicating reciprocal interactions between the ectodermal and the mesodermal layer [17]. In XLHED patients, craniofacial skeletal abnormalities including jawbone dysmorphism have frequently been reported [18]. A well-established animal model for EDA1 deficiency, the Tabby (Ta) mouse, mimicking human XLHED, results from a mutant variant of the murine gene *Ta*, which shares a 97% sequence homology with the human gene *EDA* [19,20]. In general, the Tabby phenotype reflects the characteristics of human XLHED patients, meaning that hair, teeth, and eccrine glands are affected by the developmental disorder. Tabby mice have also been reported to show spontaneous tail vertebral fractures linked to epiphyseal and subepiphyseal dysplasia [21]. *Eda*/*Edar* expression in hair follicles, teeth, and sweat glands, however, are found not only confined to the epidermis but also in neighboring dermal mesenchymal cells. In the human embryo, *EDA* expression is also not limited to the ectodermal tissues but was detected in the developing neuroectoderm, thymus, and bone [22]. In medaka fish and zebrafish, malfunctioning of the Eda pathway causes defects similar to those found in other Eda-deficient animals. Medaka nonsense mutations of its *eda* gene (all-fin less) evoke various abnormalities of the dermal skeleton, such as short and twisted fin rays, missing and abnormally shaped scales and teeth, and skull deformation, which are associated with epithelial-mesenchymal interactions [23]. The study showed that *edar* expression preceded that of the Osterix gene and that the *edar*-expressing cells migrated in the direction of fin ray elongation, indicating that the Eda/Edar signaling event precedes osteoblast differentiation. Since most of the previous studies focused on abnormalities of ectodermal appendages that are associated with Eda1 deficiency, it still remains to be elucidated whether pathogenic *Eda* variants also affect mesodermal organ development, e.g., that of the skeleton, and postnatal growth.

In a recent study [24], we focused on the pathogenesis of the characteristic kinky tail of Tabby mice and its response to prenatal treatment with an EDA1 replacement protein (Fc-EDA), consisting of the receptor-binding TNF homology domain of EDA1 and the Fc domain of human IgG1. After prenatal administration of Fc-EDA, Tabby mice showed normal tail skin proliferation and no signs of kinking, demonstrating the causal relationship between Eda1 deficiency and kinky tails. Furthermore, tibial bones in Tabby mice were found to have a higher cortical bone density than in wild-type animals, irrespective of their age, implying that Eda1 deficiency may have an influence on postnatal bone homeostasis. 

So far, only the molecular effects of Eda/Edar signaling on the development of ectodermal appendages have been elucidated. In this study, we aimed to investigate the impact of Eda1 deficiency on intramembranous bone homeostasis in adult mice, since the majority of previous studies on the role of Eda1 deficiency focused on prenatal organ development and early postnatal impact on tissues of ectodermal origin. In a previous study [24], we found that cortical bone density in long bones of Tabby mice significantly decreases with age, suggesting that Eda1 may continuously affect bone homeostasis throughout the lifetime. Since patients with ectodermal dysplasia often have craniofacial skeletal abnormalities and a recent animal study [25] also showed a predominant effect of the Ta mutation on the adult mandibular morphology via micro-CT analysis, this study focused on the intramembranous skull bone alterations in adult and old Tabby mice. Furthermore, to our knowledge, the underlying molecular mechanisms by which Eda1 influences postnatal bone homeostasis have not been reported so far, in spite of the reports of skeletal abnormalities in numerous clinical studies on patients with ectodermal dysplasia and histological/radiographic analyses in Eda1-deficient mice. Since Eda1 belongs to the TNF superfamily, which includes the osteoclast differentiating cytokine, receptor activator of nuclear factor kappa-Β ligand (RANKL), we hypothesized that Eda1 deficiency might have an impact on osteoclastic differentiation affecting the postnatal bone homeostasis, and we evaluated the localization of Eda/Edar in bone and analyzed osteoclastic activity-associated co-enzymes and transcriptional factors, such as the nuclear factor of activated T-cells, cytoplasmic 1 (NFATc1) together with nuclear factor ‘kappa-light-chain-enhancer’ of activated B-cells (NF-kB). 

Numerous studies have shown that the effects of EDA1/EDAR are largely mediated by NF-κB [26,27,28,29], although the exact mechanisms by which EDA1 regulates the development of ectodermal structures are still subject to research. The EDA1-EDAR complex recruits the receptor-associated adaptor molecule including EDARADD [30] and TNF receptor-associated factor 6 (TRAF6). TRAF6 builds a complex with TAK1 binding protein 2 (TAB2) and transforming growth factor beta-activated kinase 1 (TAK1) that activates the NF-κB essential modulator (NEMO), leading to phosphorylation and dissociation of the inhibitory complex IκBα from NF-κB [2,31,32,33]. In fact, NF-κB is also a major regulator of skeletal development and osteoclastic differentiation, requiring activation of the RANKL-RANK-TRAF6 complex [26,34,35]. *Traf6*-deficient mice or both *p50*/*p52* of NF-κB protein-inactivated mice have been reported to exhibit osteopetrosis with a lack of mature osteoclasts [34,36]. In osteoclastic differentiation, the RANKL/RANK binding activates and/or induces expression of key transcription factors, such as NF-κB, Nfatc1, cellular oncogene fos (c-Fos), microphthalmia-associated transcription factor (Mitf), and purine-rich box-1 (PU.1), which are important for osteoclastogenesis in vitro and in vivo [37,38,39,40]. Nfatc1, a master regulator of osteoclastogenesis [41], directly binds to the promoter region of osteoclast-related genes such as matrix metallopeptidase 9 (*Mmp9*), *cathepsin K*, and tartrate-resistant acid phosphatase (*Trap*), which are known to be involved in bone resorption activity [40,42,43,44]. Therefore, in the present study we investigated the localization of Eda/Edar and the underlying molecular mechanisms by which Eda1 influences bone homeostasis. 

Here, we report postnatal phenotypic changes of calvarial bone in Eda1-deficient mice and the impact of Eda1 deficiency on osteoclastic differentiation in vitro, identifying diminished nuclear Nfatc1 and bone-resorbing enzymes as a key feature that probably underlies osteopetrosis-like changes in Eda1-deficient murine calvariae.

## 2. Results

### 2.1. Osteopetrosis-like Changes of Tabby Calvarial Bone

**Figure 1:**
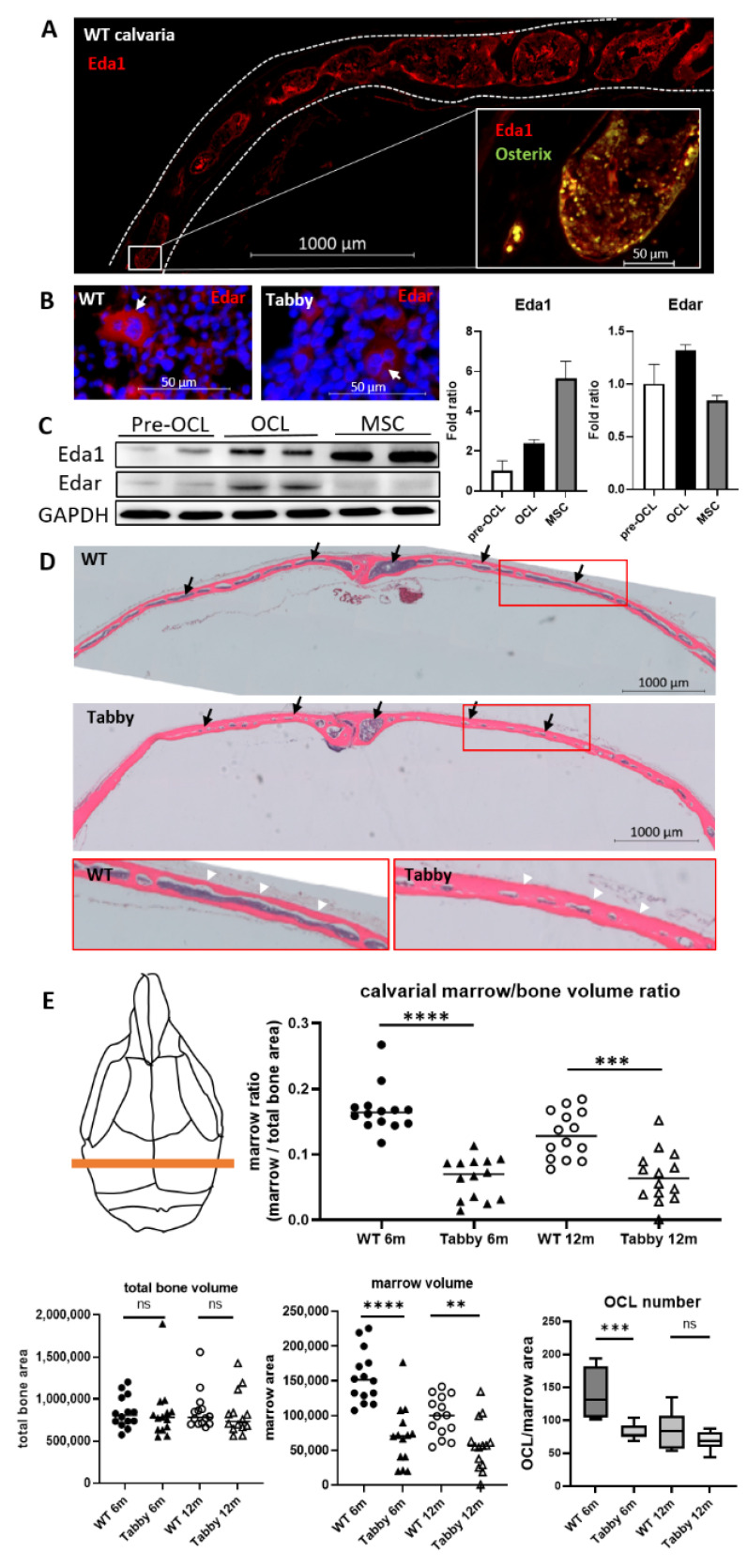
Osteopetrosis-like changes of Tabby calvarial bone. (**A**) Presence of Eda1 in 6-month-old wild-type (WT) calvariae. Dotted lines: cross-sectioned calvarial bone borders. Bar: 1 mm. Inset: co-localized Osterix+/Eda1+ endosteal bone-lining osteoblast-like cells. (**B**) Edar-presenting multinucleated osteoclasts (arrows). Edar signal is much weaker in Tabby osteoclasts compared to WT. Bar: 50 µm. Eda1 and Edar in red. Osterix in green. (**C**) Western blots for Eda1 and Edar. Pre-OCL: WT bone marrow-derived pre-osteoclasts. OCL: in vitro cultivated mature osteoclasts. MSC: rat mesenchymal stem cells. Right panel: Semi-quantitative intensity measurements from Western blots. Fold ratio with pre-osteoclast protein intensity set to 1. (**D**) Tissue sections of 6-month-old WT calvariae (representing colored zone in (**E**)). In Tabby calvariae, marrow space is strongly diminished compared to WT calvariae (black arrows), while cortical bones are thicker (white arrows). (**E**) Comparison between calvarial marrow volumes of WT and Tabby. Total bone volume (µm^2^), marrow volume (µm^2^), marrow/bone volume ratios, and endocortical osteoclast numbers in marrow area (µm^2^) were calculated for 3 consecutive sections (0.5 mm apart) of each calvaria. **, *p* < 0.01, ***, *p* < 0.001, ****, *p* < 0.0001, ns, not significant.

In this study, Eda1/Edar activity in the postnatal cranial bone was identified for the first time. Eda1 was detected immunohistochemically in the periosteum and in endosteal bone-lining cells (Osterix+/Eda1+, Figure 1A, inset) in 6-month-old wild-type (WT) calvariae (Figure 1A), while multinucleated osteoclasts proved to be Edar-presenting cells (Figure 1B). Further, the results (Figure 1C) show that preosteoclasts and osteoclasts present Eda1 as well as Osx-positive osteoblast-like cells (Figure 1A) and MSC (Figure 1C) and that mature osteoclasts have a higher Eda/Edar level than pre-osteoclasts, indicating a possible change of Eda1/Edar presence during osteoclast differentiation. This suggests that Eda may indeed be required for postnatal bone formation/resorption. 

Interestingly, we found osteopetrosis-like changes in adult Tabby (6- and 12-month-old) calvariae showing significantly reduced marrow volume (Figure 1D, arrows). This change was consistently observed in animals up to 12 months old (Figure 1E), typically accompanied by a thicker cortical bone layer in Tabby (Figure 1D, arrowheads) compared to bones of WT mice of the same age. In addition, Figure 1E shows that the total bone volumes were not different among our groups, while the marrow volume of Tabby calvariae was significantly decreased compared to that of WT calvariae. The results indicate that the apparently increased cortical bone mass is mainly due to reduced bone marrow volumes. We conclude that Eda1 deficiency results in increased endocortical bone formation and/or decreased endocortical bone resorption. Considering the reduced numbers of osteoclasts in 6-month-old Tabby calvaria compared to WT (Figure 1E), decreased endocortical bone resorption may be more relevant for increased cortical bone mass in skull bones.

Since also tibial bones of Tabby mice had a higher cortical density than in WT mice [24], we attempted to clarify whether Eda1-deficient osteoclast differentiation is related to the phenotypic changes observed in long bones and calvariae.

### 2.2. Diminished Nuclear NFATc1 and Bone-Resorbing Enzymes in Tabby-Derived Osteoclasts and Eda1-Deficient Calvariae

**Figure 2:**
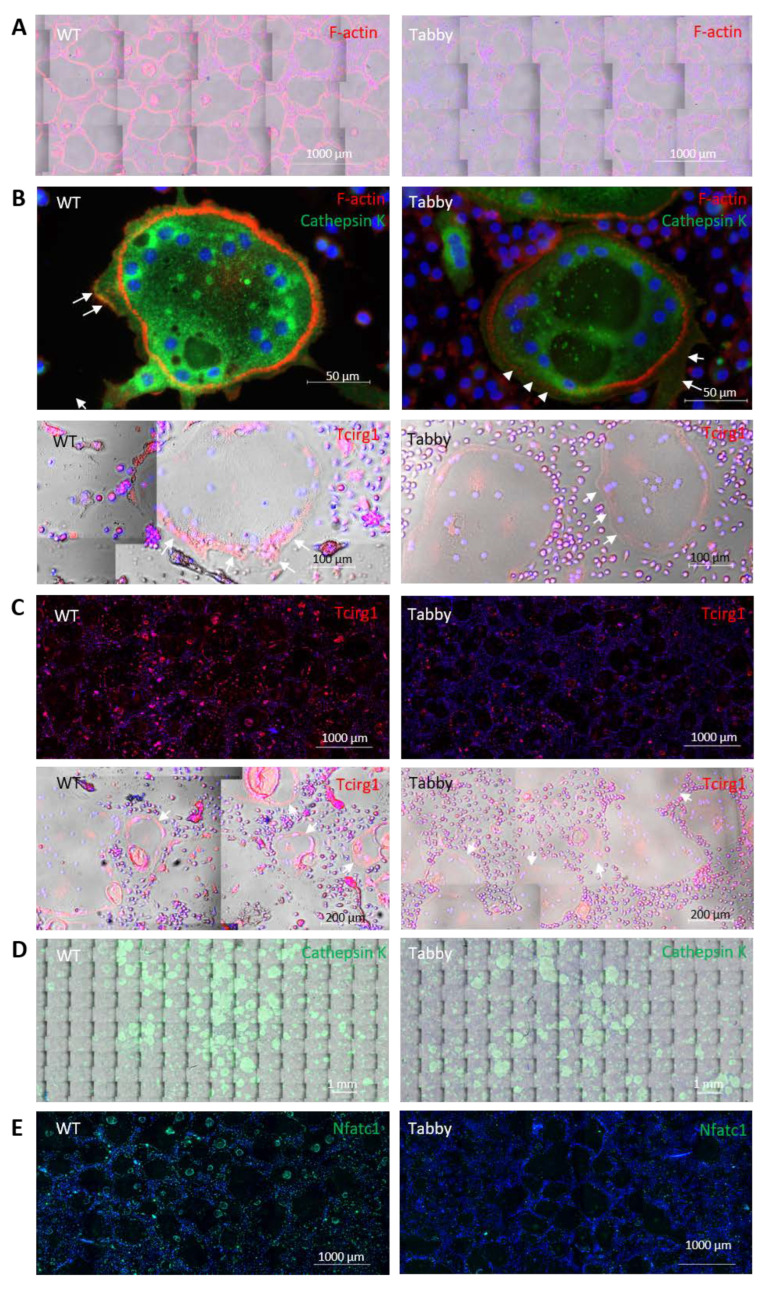

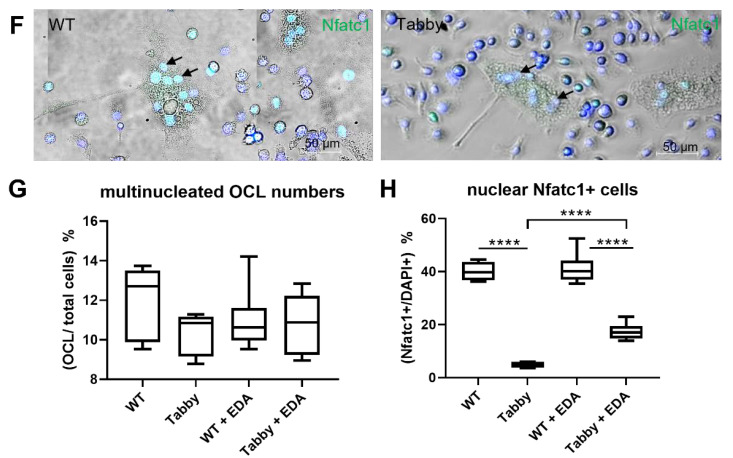
Diminished nuclear Nfatc1 and bone-resorbing enzymes in Tabby-derived osteoclasts. In vitro osteoclastic differentiation supplemented with M-CSF and RANKL for five days out of 6-week-old wild-type (WT) and Tabby bone marrow-derived mononucleated cells. (**A**) F-actin staining. (**B**) Underdeveloped and interrupted actin ring (arrowhead) with diminished cathepsin K and Tcirg1 under ruffled border (arrows) in Tabby osteoclasts compared to that in WT osteoclasts (arrows) under ruffled border. (**C**) Decreased Tcirg1 signal in Tabby osteoclasts. (**D**) Cathepsin K level was decreased in Tabby osteoclasts, but Tabby-derived differentiated osteoclast (OCL) number was not significantly altered compared to WT osteoclasts (**G**). (**D**–**H**) Fc-EDA recombinant protein supplements (50 ng/mL) during in vitro osteoclastic differentiation for four days. (**E**,**F**) Largely diminished nuclear Nfatc1 signal in mature osteoclasts of Tabby mice. (**G**,**H**) Percentage of nuclear Nfatc1+ cells was far less in Tabby osteoclasts compared to wild-type cells (**G**), while Fc-EDA-treated Tabby osteoclasts significantly recovered Nfatc1 nuclear translocation (**H**). F-actin and Tcirg1 in red. Cathepsin K and Nfatc1 in green. ****, *p* < 0.0001. n = 3 independent experiments.

Osteoclastic differentiation of bone marrow-derived mononucleated cells from either WT or Tabby mice was performed in vitro using M-CSF and RANKL supplements. However, the number of Tabby-derived differentiated osteoclasts was not significantly altered compared to the WT (Figure 2D,G), which led to the hypothesis that osteopetrosis-like changes in Tabby mice might result from diminished osteoclastic activity. A clear difference between WT and Tabby osteoclasts was found in their F-actin ring formation (Figure 2A,B), a characteristic structure that is essential for bone resorption by osteoclasts. The Tabby-derived osteoclasts often showed underdeveloped and interrupted F-actin formation (Figure 2B, arrowheads) and diminished cathepsin K and Tcirg1 in the ruffled border (Figure 2B, arrows) of Tabby osteoclasts. This finding implies that Tabby osteoclastic activities including bone resorption and cell migration may be attenuated. 

In osteoclasts, actin dynamics is associated with ATP hydrolysis and is necessary for bone resorption [45], which requires certain bone resorbing activity-associated enzymes and transcription factors. For bone resorption, actin microfilaments bind vacuolar H^+^-ATPase (V-ATPase), which locally hydrolyzes ATP for proton pumping and proton activities [46]. Among V-ATPase subtypes, variants of the *TCIRG1* gene, encoding for the osteoclast-specific a3 subunit of V-ATPase, are responsible for human recessive osteopetrosis [47] and for more than 50% of human malignant infantile osteopetrosis [48]. A recent study demonstrated that knockdown of *Tcirg1* inhibits the generation of large osteoclasts by decreasing Nfatc1 [49]. We found diminished Tcirg1 and cathepsin K (Figure 2C and D, respectively) and far fewer nuclear Nfatc1+ cells in Tabby mature multinucleated osteoclasts (Figure 2E,F arrows). The concomitant downregulation of Tcirg1 and nuclear Nfatc1 implies that Tabby mature osteoclasts may be deficient in any function essential for bone-resorbing activity.

To clarify whether Eda1 deficiency is responsible for the reduced Nfatc1 nuclear translocation, Fc-EDA was added during in vitro Tabby mature osteoclastic differentiation. Fc-EDA-treated Tabby osteoclasts showed significantly increased nuclear Nfatc1, suggesting that Eda1 may enhance Nfatc1 transcriptional activity during late osteoclastic differentiation (Figure 2H).

**Figure 3:**
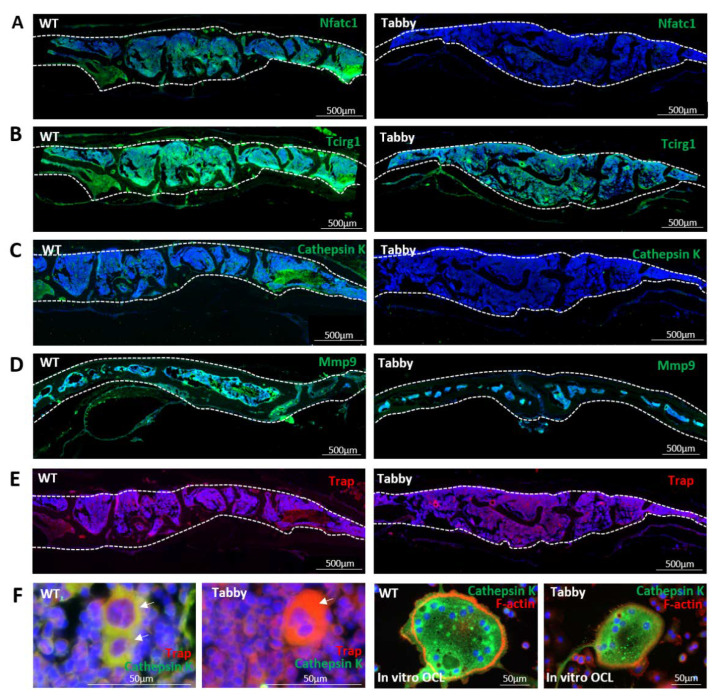
Significantly decreased NFATc1 and bone-resorbing enzymes in Eda1-deficient calvariae. Immunostainings for Nfatc1 and bone-resorbing enzymes in 12-month-old wild-type (WT) and Eda1-deficient Tabby calvariae. Dotted lines: cross-sectioned calvarial bone borders. (**A**–**D**) Strongly decreased Nfatc1, Tcirg1, cathepsin K, and Mmp9 in Tabby calvariae, while Trap in Tabby calvaria was comparable with that in WT (**E**). (**F**) Left panels: co-stained Trap/Cathepsin K in WT calvarial osteoclasts in contrast to Trap^bright^/Cathepsin K^dim^ Tabby calvarial osteoclasts. Right panels: in vitro differentiated Tabby-derived osteoclasts also show Cathepsin K^dim^ signal. Nfatc1, Tcirg1, Cathepsin K, and Mmp9 in green. Trap and F-actin in red.

Based on the preceding in vitro results indicating the diminished resorption-associated co-enzymes in Tabby-derived osteoclasts, the corresponding activities in intramembranous bones, particularly in calvariae (Figure 3), were investigated. In line with the in vitro results, immunofluorescence staining of Tabby calvariae revealed strongly diminished Nfatc1 and Tcirg1 signals (Figure 3A,B). Cathepsin K and Mmp9, two enzymes encoded by target genes of NFATc1 transcriptional activation and both involved in bone remodeling and resorption, were also significantly decreased in Tabby bone (Figure 3C,D), whereas Trap activity was comparable with WT levels (Figure 3E). The latter is in line with our previous in vivo results showing that the numbers of Trap-positive cells in Tabby tibiae trabeculae were comparable with trabeculae of WT mice of the same age [24]. Tabby calvarial osteoclasts presented Trap^bright^/cathepsin K^dim^ staining in a double immunostaining, whereas WT osteoclasts showed cathepsin K^bright^/Trap^bright^ stainings (Figure 3F, left panels). This finding was further confirmed by in vitro differentiated Tabby bone marrow-derived osteoclasts showing a diminished cathepsin K and F-actin ring (Figure 3F, right panels). 

**Figure 4:**
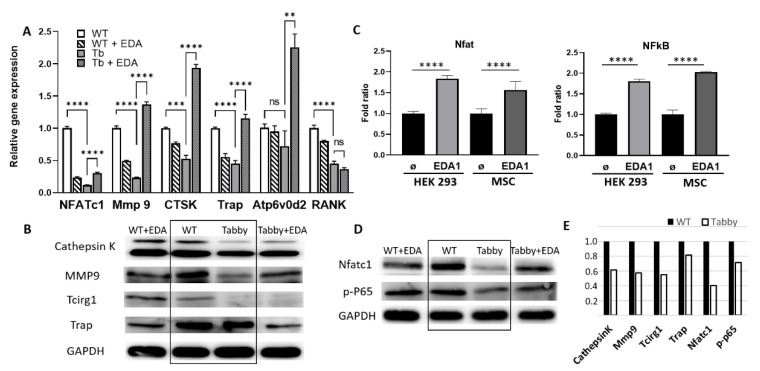
EDA1 induces NF-kB and Nfatc1 activation in osteoclastic differentiation. Quantitative RT-PCR (**A**), Western blots (**B**), and luciferase assays (**C**) using in vitro differentiated osteoclasts supplemented with M-CSF and RANKL either in the presence or the absence of Fc-EDA (50 ng/mL) for five days out of 6-week-old wild-type (WT) and Tabby bone marrow-derived mononucleated cells. **, *p* < 0.01, ****, *p* < 0.0001, ns, not significant. (**D**,**E**) Western blots. Cells were harvested 10 min after Fc-EDA (50 ng/mL) treatment. (**E**) Semi-quantitative intensity measurements from Western blots (**B,D**). Fold ratio with WT protein intensity set to 1.

Immunohistochemical staining indicates that diminished signals for Nfatc1, Tcirg1, cathepsin K, and Mmp9 in skull bones are associated with decreased number and/or activity of Tabby osteoclasts. Hence, we further investigated the expression of resorbing activity-associated genes or their protein levels in WT and Tabby osteoclasts and their transcription factor activations via quantitative RT-PCR, Western blot, and luciferase assays (Figure 4).

### 2.3. EDA1 Induces NF-kB and Nfatc1 Activation in Osteoclastic Differentiation

Quantitative RT-PCR (Figure 4A) and Western blot (Figure 4B) revealed downregulated gene expression as well as diminished protein levels of cathepsin K, Mmp9, and Tcirg1 in Tabby-derived differentiated osteoclasts, which is in good agreement with our in vitro (Figure 2) and in vivo results (Figure 3).

Notably, significantly decreased expression of *Mmp9*, *Ctsk* (*cathepsin K*), *Trap*, and *ATP6v0d2* (V-ATPase d2 subtype; together with *Tcirg1* one of the major subtypes in osteoclasts) were completely recovered via Fc-EDA supplementation during differentiation in Tabby-derived osteoclasts (Figure 4A), whereas diminished *Nfatc1* expression was not altered as much by Fc-EDA. Considering that *Ctsk*, *Mmp9*, and *Trap* are transcriptional target genes of Nfatc1 [40,42,44], this finding may indicate EDA1 as an inducer of Nfatc1-mediated transcriptional activation of those genes. We cannot exclude the possibility that Fc-EDA treatment may affect the post-transcriptional translation or post-translational modification of proteolytic enzymes. It is noteworthy that gene expression levels in WT osteoclasts treated with Fc-EDA were rather decreased compared to WT osteoclasts in the absence of Fc-EDA treatment, suggesting a further time-lapse study to elucidate the possibility of a negative feedback loop triggered by excessive EDA1 levels in WT cells. The protein levels of cathepsin K, Mmp9, and Trap as well as Tcirg1 in Eda1-deficient Tabby osteoclasts were generally diminished compared to those in WT osteoclasts (Figure 4B,E). Thus, the osteopetrosis-like phenotype in vivo might be attributed to the diminished osteoclastic activity possibly due to Eda1 deficiency. Interestingly, cathepsin K and Mmp9 levels in Tabby osteoclasts were partially rescued by Fc-EDA treatment, while Trap protein levels in WT osteoclasts following supplementation of Fc-EDA were decreased similar to the gene expression change in Fc-EDA-treated WT osteoclasts. Overall, data from quantitative RT-PCR and Western blot clearly suggest that osteoclastic activity-associated gene expressions and their protein levels sensitively respond to Eda1 presence. Apart from osteoclastic activity-associated gene expression, the results show in particular that the expression of RANK (Figure 4A) was reduced in Tabby-derived osteoclasts compared to that in WT cells, while diminished RANK expression in Tabby cells was not restored by Fc-EDA treatment. Considering that RANKL/RANK as well as Eda1 belongs to the TNF superfamily and in vitro cultivated cells were stimulated by RANKL, the diminished *RANK* expression in Tabby osteoclasts would result in decreased numbers of osteoclasts in cultures stimulated by RANKL. These data (Figure 4A and Figure 1E) indicate the possibility that Eda1 deficiency may affect both the formation of osteoclasts and their activity at least in part via the diminished *RANK* expression, which might also affect the transcriptional activation of Nfatc1 and NF-κB as well as the expression of Nfatc1 in Tabby osteoclasts (Figure 4A).

Further we wondered whether Eda1 is transferred to its downstream signaling through Nfat and NF-κB pathways and hypothesized that it may trigger the NF-κB downstream signaling pathway not only in ectodermal appendages but also during osteoclastic differentiation. Luciferase assays were performed by adding Fc-EDA either to the HEK 293 cell line or to rat mesenchymal stem cells (MSCs) following transfection with NFAT-RE-Luc and NF-κB-Luc luciferase reporter plasmids. The results showed that both Nfat and NF-κB transcriptional activation were elevated in cells of epidermal as well as mesenchymal origin, responding to EDA1 stimulation (Figure 4C). 

In order to identify whether EDA1 could induce the phosphorylation of the NF-κB p-65 subunit, we supplemented Fc-EDA 10 min before harvesting osteoclasts differentiated with RANKL and M-CSF for four days. Of note, NF-κB phospho-p-65 and Nfat protein levels in Tabby-derived osteoclasts were considerably recovered via Fc-EDA treatment (Figure 4D). In contrast, untreated Tabby-derived osteoclasts showed dramatically reduced NF-κB phospho-p-65 and Nfat protein levels compared to WT-derived cells (Figure 4E). This indicates that EDA1 might be one of the ligands or stimulants, such as TNF-α, IFN-γ, or RANKL, that trigger downstream NF-κB and NFAT signaling in osteoclast development.

**Figure 5:**
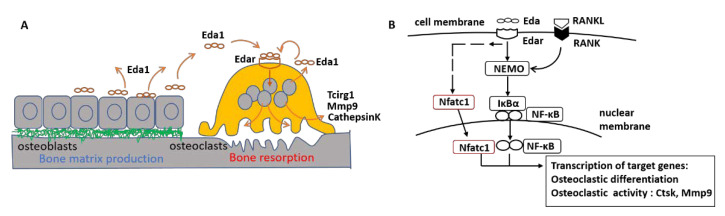
A hypothetical model of Eda1/Edar-regulated bone homeostasis. Based on our in vitro and in vivo results, a hypothetical model suggests that Eda1/Edar interactions between EDA-presenting osteoblasts and Edar-presenting osteoclasts may be a relevant communicational signal enabling concerted postnatal bone homeostasis (**A**) and that Eda1 induces Nfat and/or NF-κB transcriptional activation, leading to the expression of osteoclastic activity-associated genes, such as *Ctsk*, *Mmp9*, *Trap*, and *Tcirg1* (**B**). Inversely, Eda1 deficiency in mice may result in diminished osteoclastic activity, resulting in osteopetrosis-like changes and causing a disturbed intramembranous bone homeostasis during a postnatal period.

The present findings in vitro and in vivo suggest that Eda1-deficiency in mice provokes a disturbed intramembranous bone homeostasis leading to osteopetrosis-like changes, possibly associated with the diminished osteoclastic activity. Moreover, Eda1 may play an important functional role in postnatal bone growth and lifelong homeostasis, potentially via the Nfat and/or NF-κB signaling pathway (Figure 5).

## 3. Discussion

This study provides the first molecular approach to reveal the role of Eda1 in bone homeostasis, also confirming previous observations that Eda1 deficiency leads to skeletal impairments reported by our and other groups [21,24,25]. Hill et al. showed that adult Tabby mice display spontaneous fractures of vertebral bodies in the caudal spine linked to epiphyseal and subepiphyseal dysplasia [21]. Bornert et al. also reported morphological changes in the mandible of Tabby mice detected via micro-CT imaging analysis [25]. In a previous study, we found that higher cortical bone density in Tabby long bones is maintained postnatally irrespective of age [24]. Consistent with these findings, we observed reduced marrow volume and a thicker cortical bone layer in Tabby calvariae compared with WT. Recent studies indicate that osteopetrosis is associated with severe clinical abnormalities such as increased bone density and lack of bone marrow cavity [50]. Thus, more detailed complete analysis of changes in cortical bone mass and decreased bone marrow volumes and width in skull bones, vertebrae and long bones of Tabby mice compared with WT mice focusing mainly on the quantitative assessments of cortical and trabecular bone will be part of a follow-up project. These investigations may provide valuable experimental evidence of the specific role of Eda1 in bone homeostasis and potential downstream target molecules such Nfac1, Tcirg1, Ctsk, or Mmp9. 

Our previous findings and the results presented here suggest that Eda1 deficiency entails intramembranous and endochondral bone defects in mice. To our knowledge, this is the first report about the localization of Eda1/Edar in postnatal bones and demonstrates the osteopetrosis-like changes in Eda1-deficient adult intramembranous bones of the skull. In particular, we focused on revealing the functional role of Eda1 and verifying its downstream signaling pathways. The results show that Eda1 affects osteoclastic differentiation in postnatal bone maintenance, especially by regulating gene expressions and transcriptional activations associated with bone-resorbing activity. Presumably, osteoclasts may not be the sole Eda1/Edar target cell type in skeletal systems. Indeed, bone lining-osteoblast-like Osterix+ cells produce and present Eda1 (Figure 1). Mesenchymal stem cells and osteoblastic lineages may also be affected by phenotypic changes due to Eda1 deficiency. A previous paper speculated that *Eda1* expression in osteoblasts might be a functionally important factor in bone development [21]. Our current results confirm the presence of Eda1/Edar in bone and suggest that, in addition to their well-known role in reciprocal epithelial–mesenchymal interactions in ectodermal appendages, Eda1/Edar interactions between Eda-presenting osteoblasts and Edar-presenting osteoclasts might be a relevant communicational signal enabling concerted postnatal bone homeostasis with these two types of cells. Further in vivo studies will be needed to investigate long-term effects of postnatal recombinant EDA1 administration on skeletal growth and maintenance both in normal and Eda1-deficient animal models. Of note, in contrast to the findings of increased cortical bone density in Tabby long bones [24] and skull bones in the present study, cortical bone density in vertebral bones of the kinky tail appeared to be comparatively low in Tabby mice aged 2–4 months and was significantly reduced in older Tabby mice [24]. Yet, the molecular mechanisms causing the differential anatomical site-dependent skeletal abnormalities are not clear. In the literature, numerous studies have proposed a role of Eda1 in reciprocal epithelial–mesenchymal interactions. One of the hypotheses could be that skeletal alterations in Tabby mice may be affected by neighboring defective ectodermal growth and development. Our previous study [24] also suggested that disturbed bone development in kinky tails is due to skin devoid of hair follicles which does not grow fast enough to accommodate the incessantly growing caudal vertebral bodies. Further investigations to identify the role of Eda1 in the reciprocal epithelial–mesenchymal interactions surrounding a bone region may elucidate differential influences of Eda1 deficiency on bone homeostasis according to anatomical locations and developmental patterns.

The present study demonstrated that Nfatc1-mediated transcriptional activation is triggered by Fc-EDA, indicating a novel downstream signaling pathway of Eda1 in osteoclastic differentiation. EDA/EDAR signaling has been reported to be linked with RANKL and TRAF6 via the NF-κB pathway [26,27,28]. In fact, the RANKL/TRAF6/NF-κB pathway is a well-known classical signaling pathway for osteoclastic differentiation [26,34,35]. NFAT signaling also plays a critical role in osteoclastic maturation and bone-resorbing activity [40,41]. In the current study, the concomitant decrement of the osteoclastic activity-associated transcription factor and enzymes in the Tabby mouse may suggest that Eda1/Edar plays a so far unrevealed role in osteoclastic differentiation processes and osteoclastic activation. Fc-EDA-induced Nfatc1 activation, followed by elevated *Ctsk*, *Mmp9*, and *Trap* expressions (known as Nfatc1-transcriptional target genes) indicates that Eda1/Edar/RANKL may deliver their interacting signals through the Nfat as well as NF-κB pathway. We cannot rule out that the regulatory mechanisms for these enzymes could be different. In particular, regulation of *Trap* expression seems to be complex. It has been reported to be regulated at the transcriptional level not only by Nfat but also by Mitf, and the interaction between Mitf and Nfat might be crucial as well [51,52]. In addition, Trap expression appears to be regulated by several alternative tissue- and cell-restricted promoters, and *Trap* mRNA stability might be controlled post-transcriptionally [53]. Yet, it remains to be elucidated by which dimers of NF-κB subunits Eda1 efficiently affects osteoclastic activation via canonical or non-canonical NF-κB signaling pathways. Moreover, it is still unclear based on our in vitro results why the number of Tabby-derived osteoclasts was not different from that of WT osteoclasts. Considering the crucial role of Nfatc1 as a transcription factor for osteoclast progenitor cell differentiation and osteoclast formation, a decrease of Nfatc1 should result in reduced osteoclast formation. To confirm the impact of Eda1 on osteoclast formation and activity, further studies should include experiments with RANKL-stimulated cells from these mice on bone slices to assess ex vivo osteoclast numbers and resorption activity under more in vivo-like conditions. In addition, we cannot exclude the possibility that Eda1, with yet unrevealed interaction partners, affects Nfatc1 transcriptional activation differently depending on the differentiation stage of osteoclasts or that *Eda1* expression may be regulated according to the differentiation stages. The regulatory mechanisms of Eda1 at different osteoclastic differentiation stages and the regulation of *Eda1* expression at different differentiation stages are certainly worth investigating. Furthermore, to confirm that Eda1 deficiency has an impact on osteoclastic activity, experiments verifying the osteoclastic resorption activity and quantification of serum markers for bone resorption/formation in Tabby-derived osteoclasts and Tabby bones as well as dynamic histomorphometry to assess bone formation will be worth performing in a future study. 

In addition, it has been well documented that Eda1 interacts with many other important partners of morphogens, such as Wnts, BMPs, and hedgehogs, during ectodermal organ development. Potentially, these interacting partners may also play critical roles in pre- and postnatal skeletal growth and development, collaborating with Eda/Edar in skeletal systems. In fact, in the Tabby mouse, Eda1 deficiency did not result in dramatic deteriorations in prenatal and early postnatal skeletal development. Our results rather implicate that Eda/Edar may contribute to a postnatal fine-tuning, compensating for or accelerating demands for juvenile bone growth and adult bone homeostasis via those interacting partners. Regarding the persistent presence of Eda1 throughout the lifetime in murine bones, future work may reveal that Eda1 is required for life-long physiologic functions in skeletal systems.

In conclusion, this study showed the impact of Eda1 deficiency on postnatal calvarial bone homeostasis. Though craniofacial abnormalities are commonly found in hypohidrotic ectodermal dysplasia patients and a direct role of Eda and underlying molecular aspects in the development of the craniofacial complex is still elusive, the present results may deepen the basic understanding of EDA1-related molecular mechanisms in postnatal craniofacial bone growth.

## 4. Materials and Methods

### 4.1. Animal Model

C57BL/6 wild-type mice (Charles River, Sulzfeld, Germany), white-bellied agouti B6CBAa Aw-J/A-EdaTa/J Tabby mice (a mouse model for XLHED; Jackson Laboratory, Bar Harbor, ME, USA), and appropriate control mice were housed in individually ventilated cages under standard conditions with a light/dark cycle of 12 h and free access to standard chow and tap water. All experimental procedures on animals were conducted in accordance with German regulations and legal requirements and had been approved by the local government authorities.

### 4.2. Histological Assessments and Calvarial Bone Histomorphometry

For histological assessments, calvarial bone specimens of 6-, 9-, 12-month-old adult mice were fixed with 4% paraformaldehyde and decalcified in 14% (wt/vol) EDTA (pH adjusted to 7.2 by addition of ammonium hydroxide) for 14 days and then embedded in paraffin. Calvarial bones were cross-sectionally cut into 10 µm thickness, and tissue sections were stained with hematoxylin and eosin. For histomorphometric analysis, cross-sectioned consecutive sections (0.5 mm apart, 3 different areas of sections per calvaria) in the middle of the sagittal suture area (see Figure 1C) were examined. Tissue sections were stained with hematoxylin and eosin to visualize cortical bone and marrow space. Total bone area, marrow space, and their ratio were calculated via digital image analysis (Zen 3.1, Axio Observer 7, Zeiss, Jena, Germany).

### 4.3. In vitro Cell Culture and Osteoclast Differentiation

For cell isolation and in vitro differentiation, mononucleated cells were isolated from 6-week-old tibia bone marrow of Tabby and C57BL/6 wild-type mice. For osteoclast differentiation, freshly isolated whole bone marrow mononucleated cells were plated with α-MEM, 10% fetal calf serum (FCS, PAN biotech, Aidenbach, Germany), L-glutamine, penicillin, and streptomycin (Sigma-Aldrich, Burlington, MA, USA). The next day, non-adherent cells were collected and newly plated with 10 ng/mL M-CSF for two days followed by differentiation culture for five days with 50 ng/mL mouse RANKL (Immunotools, Friesoythe, Germany) and 50 ng/mL M-CSF (Immunotools). During the differentiation culture period, half of the culture medium was changed every two days with 50 ng/mL Fc-EDA treatment. The osteoclasts differentiated for five days were used for immunofluorescence staining, quantitative RT-PCR, and Western blot analysis. For Western blot analysis of Eda and Edar, mononucleated cells isolated from 6-week-old tibia bone marrow of Tabby and C57BL/6 wild-type mice were further positively selected by CD14 antibody (Biolegend, San Diego, CA, USA) using a MACS separator (Miltenyi Biotec, Bergisch Gladbach, Germany) to evaluate the protein levels of osteoclasts cultivated from relatively homogenous monocytes/macrophage populations excluding the mixed cultivation with stromal cells or other lineage-derived cells from isolated bone marrow cells. The CD14-positive cells were cultivated with 50 ng/mL mouse RANKL (Immunotools) and 50 ng/mL M-CSF (Immunotools) for two days for pre-osteoclasts or five days for mature osteoclasts for Western blot analysis.

For luciferase assays, rat mesenchymal stem cells were isolated and expanded from fresh bone marrows from femurs of 4-week-old Wistar rats as described previously [54]. Selected clonal cells were further expanded in medium containing 60% DMEM-LG (Invitrogen, Waltham, MA, USA), 40% MCDB-201 (Sigma-Aldrich), and supplemented with 1× insulin-transferrin-selenium (Sigma-Aldrich), 1× linoleic acid-bovine serum albumin (Sigma-Aldrich), 10^−9^ M dexamethasone (Sigma-Aldrich), 10^−4^ M ascorbic acid 2-phosphate (Sigma-Aldrich), 100 units of penicillin, 1000 units of streptomycin (Invitrogen), 10 ng/mL EGF (Sigma-Aldrich), 10 ng/mL PDGF-BB (Immunotools), 1000 units/mL of rat LIF (Sigma-Aldrich), and 2% FCS. The expanded cells showed the potential to differentiate into multiple mesenchymal lineages including osteoblasts, chondroblasts, adipocytes, myoblasts, myofibroblasts, and endothelial cells in differentiation experiments in vitro [55]. The expanded MSCs were further cultivated with α-MEM, 10% FCS, penicillin and streptomycin, the HEK 293 cells with DMEM (Invitrogen), 10% FCS, penicillin, and streptomycin.

### 4.4. Immunostaining and Acquisition of Images

For immunofluorescence, 10 μm calvarial tissue slides subjected to antigen retrieval in citrate buffer and in vitro cultivated cells fixed with 4% paraformaldehyde were permeabilized via incubation with 0.2% Triton X-100 in PBS for 10 min and washed with PBS. Primary antibodies used were: rabbit anti-Eda1(Thermo Fisher Scientifics, Waltham, MA, USA), anti-Osterix (Abcam, Cambridge, UK), anti-Edar (MyBioSource, San Diego, CA, USA), anti-F-actin (Thermo Fisher Scientifics), anti-Tcirg1 (Thermo Fisher Scientifics), anti-Mmp9 (Sigma-Aldrich), anti-Trap (Abcam), anti-phospho-p65 (Cell signaling, Danvers, MA, USA), mouse anti-Eda1 (EctoD2 and EctoD3, generated by Kowalczyk-Quintas, et al. [56]), anti-Nfatc1 (Sigma-Aldrich), anti-Cathepsin K (Abcam), anti-GAPDH (Thermo Fisher Scientifics) and antibodies diluted in antibody diluent (DAKO, Copenhagen, Denmark) in overnight incubation at 4 °C. Probes were washed with PBS two times followed by incubation with fluorochrome-conjugated secondary antibodies. Staining in the absence of primary antibodies confirmed the specificity of the immunolabeling. Fluorescence was monitored using an Axio Observer 7 microscope (Zeiss). For analyzing nuclear Nfatc1, osteoclastic differentiation was performed for four days with 50 ng/mL mouse RANKL (Immunotools) and 50 ng/mL M-CSF (Immunotools) out of freshly isolated wild-type and Tabby bone marrow-derived mononucleated cells. During the differentiation culture period, half of the culture medium was changed every two days with 50 ng/mL Fc-EDA treatment. After immunostaining for Nfatc1, nuclear Nfatc1-positive multinucleated cells (nuclei ≥ 3) were counted in three independent culture dishes for each group. The ratio of multinucleated osteoclasts or nuclear Nfatc1-positive multinucleated cells among the total number of cells was calculated (Figure 2B,C). 

### 4.5. Quantitative Real-Time PCR

Total RNA was extracted from differentiated osteoclasts as described in the in vitro cell culture section above using TRIzol™ reagent (Thermo Fisher Scientific) and a standard protocol for RNA isolation. cDNA was synthesized via reverse transcription PCR with the Moloney Murine Leukemia Virus Reverse Transcriptase system (Promega GmbH, Mannheim, Germany) according to the manufacturer’s instructions. The iQ™ SYBR^®^ Green Supermix (Bio-Rad Laboratories GmbH, Munich, Germany) and exon-exon spanning primers avoiding genomic DNA amplification were used for quantitative real-time PCR (RT-PCR; primer sequences and thermal cycling conditions are available upon request). The gene encoding the 18S ribosomal RNA (rRNA) was used as a housekeeping gene for normalization. All measurements were performed in quadruplicate and averaged. Relative gene expression levels were calculated using the delta-delta Ct method.

### 4.6. Western Blot

Soluble cytosolic proteins were extracted from differentiated osteoclasts with lysis buffer (150 mM NaCl, 50 mM Tris pH 7.4, 1% NP-40, 0.25% deoxycholate and 1 mM EDTA, PMSF 1 mM, NaF 1 mM, Na3VO4 1 mM, 1 μM of aprotinin, leupeptin, pepstatin) for 10 min at 4 °C. After centrifugation at 13,000× *g* for 20 min at 4 °C, protein lysates were denatured using a loading buffer with the anionic detergent sodium dodecyl sulfate at 96 °C for 5 min, separated using 10% SDS-PAGE, and electroblotted onto a PVDF membrane (Roti-PVDF; Roth GmbH, Karlsruhe, Germany) according to standard protocols. After blocking in 5% nonfat dry milk/TBST containing 0.1% Tween for 2 h, membranes were incubated overnight at 4 °C with rabbit anti-Tcirg1 (Thermo Fisher Scientifics), anti-Mmp9 (Sigma-Aldrich), anti-Trap (Abcam), anti-phospho-p65 (Cell signaling), anti-Eda (Invitrogen), mouse anti-Nfatc1 (Sigma-Aldrich), anti-cathepsin K (Abcam), anti-Edar (R&D Systems, Minneapolis, MA, USA), and anti-GAPDH (Thermo Fisher Scientifics) antibodies, diluted in 2% BSA/TBST buffer. Membranes were then washed with TBST buffer solution, incubated with horseradish peroxidase-conjugated secondary antibody (dilution 1:500; Cell signaling), and proteins were detected using enhanced chemiluminescence (ECL; Sigma-Aldrich, Taufkirchen, Germany). Equal protein loading was ensured by probing blots with an anti-GAPDH antibody. For semi-quantitative comparison of the groups, the intensity of acquired protein bands was measured using ImageJ software (NIH, Bethesda, MD, USA) and their fold ratio was calculated with the WT protein intensity set to 1 (Figure 4E).

### 4.7. Luciferase Assays

Transfection of HEK293 cells or MSCs was performed with the K4 transfection system (Biontex, Martinsried, Germany) as recommended by the manufacturer. Cells were plated one day earlier for transfection in each 6-well plate (1.5 × 10^5^) with 2 mL of cell culture medium specified in the in vitro culture section above. Transfections were performed with NFAT-dependent reporter plasmid pGL3/NFAT-RE-Luc (Promega) or NF-κB-dependent reporter gene vector pBIIX-Luc (Promega), 1 μg each for HEK293 cells and MSCs. Each transfection was carried out in triplicate. Fc-EDA (50 ng/mL) was treated two hours before measurement and luciferase activities were measured 24 h after transfection using the Luciferase Assay System (Promega), and relative light units were measured using a Veritas luminometer (Promega, Turner Biosystems, Sunnyvale, CA USA).

### 4.8. Statistical Analyses

Individual values from independent experiments were summarized as means plus standard deviations. Differences between mean values from the datasets were analyzed with unpaired t-tests using the GraphPad Prism software 9 (GraphPad Software Inc., La Jolla, CA, USA).

## Data Availability

The datasets used and analyzed during the study are available from the corresponding author upon reasonable request.
